# Genetic analysis of *Caenorhabditis elegans pry-1/Axin* suppressors identifies genes involved in reproductive structure development, stress responses, and aging

**DOI:** 10.1093/g3journal/jkab430

**Published:** 2021-12-15

**Authors:** Avijit Mallick, Nikita Jhaveri, Jihae Jeon, Yvonne Chang, Krupali Shah, Hannah Hosein, Bhagwati P Gupta

**Affiliations:** Department of Biology, McMaster University, Hamilton, ON L8S4K1, Canada

**Keywords:** *C. elegans*, *pry-1*, Axin, vulva development, stress response, aging, WNT signaling

## Abstract

The Axin family of scaffolding proteins regulates a wide array of developmental and post-developmental processes in eukaryotes. Studies in the nematode *Caenorhabditis elegans* have shown that the Axin homolog PRY-1 plays essential roles in multiple tissues. To understand the genetic network of *pry-1*, we focused on a set of genes that are differentially expressed in the *pry-1*-mutant transcriptome and are linked to reproductive structure development. Knocking down eight of the genes (*spp-1, clsp-1, ard-1, rpn-7, cpz-1, his-7, cdk-1*, and *rnr-1*) via RNA interference efficiently suppressed the multivulva phenotype of *pry-1* mutants. In all cases, the ectopic induction of P3.p vulval precursor cell was also inhibited. The suppressor genes are members of known gene families in eukaryotes and perform essential functions. Our genetic interaction experiments revealed that in addition to their role in vulval development, these genes participate in one or more *pry-1*-mediated biological events. Whereas four of them (*cpz-1, his-7, cdk-1*, and *rnr-1*) function in both stress response and aging, two (*spp-1* and *ard-1*) are specific to stress response. Altogether, these findings demonstrate the important role of *pry-1* suppressors in regulating developmental and post-developmental processes in *C. elegans.* Given that the genes described in this study are conserved, future investigations of their interactions with Axin and their functional specificity promises to uncover the genetic network of Axin in metazoans.

## Introduction

Most genetic research is aimed at linking genes to phenotypes and understanding how changes in gene function affect biological processes. Studies in animal models have demonstrated that genes exert their effects through interactions with other genes that form functional networks. Disruptions of the activity of network components can lead to various defects and in some cases premature death. Therefore, a comprehensive understanding of gene–gene interactions is crucial for the discovery of effective treatments. Our group is currently investigating the genetic network of an Axin family member in the nematode *Caenorhabditis elegans*. Axins are scaffolding proteins that play crucial roles in regulating conserved processes in metazoans; they integrate inputs from multiple interactors to coordinate downstream cellular signaling events. Moreover, Axin mutations have been implicated in multiple abnormalities and diseases (Mallick *et al.* 2019b). Therefore, elucidating the Axin signaling cascade can enhance our understanding of disease progression, and the pathway could be an attractive therapeutic target.

Work from our lab and others have shown that the *C. elegans* Axin homolog PRY-1 is involved in multiple developmental and post-developmental processes, including embryogenesis, neuronal differentiation, vulva formation, seam cell development, lipid metabolism, and lifespan maintenance (Maloof *et al.* 1999; Korswagen *et al.* 2002; Mallick *et al.* 2019b, 2020; Mallick and Gupta 2020). Initial studies on *pry-1* showed that the gene product acts as a negative regulator of canonical WNT signaling (Korswagen *et al.* 2002). PRY-1 forms a destruction complex in the absence of a WNT ligand, leading to inhibition of a WNT effector, the β-catenin homolog BAR-1. Mutations in *pry-1* mimic activated WNT signaling and cause the translocation of BAR-1 to the nucleus, thereby promoting the expression of target genes (Gleason *et al.* 2002). During vulval development, *pry-1* restricts the number of induced vulval precursor cells (VPCs). In a normal worm, three (P5.p, P6.p, and P7.p) of the six Pn.p cells (P3.p through P8.p), termed the VPCs, participate in the formation of the vulva (Sulston and Horvitz 1977; Sternberg 2005). The remaining uninduced VPCs adopt nonvulval fates and fuse with the surrounding hypodermal syncytium (Sternberg and Horvitz 1989). In the absence of *pry-1*, nonvulval cells are inappropriately induced to adopt specific fates, resulting in multiple ectopic ventral protrusions, a phenotype termed multivulva (Muv) (Gleason *et al.* 2002; Seetharaman *et al.* 2010). The mechanism of *pry-1* action during VPC induction and cell fate specification is not well understood.

In addition to its essential function in canonical WNT signaling, *pry-1* participates in the WNT asymmetric pathway to regulate the expression of heterochronic microRNAs and their targets during seam cell development (Mallick *et al.* 2019a). More recently, we discovered additional roles for *pry-1* in the regulation of lipid metabolism, stress response, and aging, and identified its interacting genes in these processes (Mallick *et al.* 2019b, 2020). Whereas these findings demonstrate the essential function of *pry-1* in *C. elegans*, how it controls diverse events and mediates specific cellular interactions remain to be explored. Given that the gene encodes a scaffolding protein, it might recruit many other factors to regulate their activities.

In this study, we set out to investigate the genetic network of *pry-1* by investigating a set of genes that are involved in a wide variety of cellular and molecular processes. The genes are differentially expressed in the *pry-1*-mutant transcriptome and while they are all associated with the GO term “reproductive structure development,” many have essential roles in other tissues as well. Our RNAi knockdown experiments revealed that eight of the 26 genes tested (*spp-1*, *clsp-1*, *ard-1*, *rpn-7*, *cpz-1*, *his-7*, *cdk-1*, and *rnr-1*) strongly suppressed the *pry-1* Muv phenotype and ectopic P3.p induction. We examined genetic interactions between the genes and *bar-1* in vulval cells, which revealed that *rpn-7* acts downstream of *pry-1-bar-1*-mediated signaling. All of the suppressor genes are conserved in eukaryotes and perform diverse functions such as oxidation-reduction reactions (dehydrogenase family: *ard-1* and reductase family: *rnr-1*), protein degradation (proteasomal complex component: *rpn-7* and peptidase: *cpz-1*), protein phosphorylation (adapter protein: *clsp-1* and kinase: *cdk-1*), transmembrane transportation (channel protein: *spp-1*), and the regulation of gene expression (histone: *his-7*) (Davy *et al.* 2001; Hashmi *et al.* 2004; Boxem 2006; Alper *et al.* 2007; Mori *et al.* 2008; Srinivasan *et al.* 2008; Ossareh-Nazari *et al.* 2016). Four of the genes (*clsp-1*, *his-7*, *cdk-1*, and *rnr-1*) have roles in the cell cycle, DNA damage checkpoint, DNA repair, and DNA replication.

We investigated whether suppressors also participate in other *pry-1*-mediated processes, such as the stress response and lifespan maintenance. Whereas RNAi knockdown of four of the eight genes (*cpz-1*, *his-7, cdk-1*, and *rnr-1)* significantly rescued the short lifespan and stress sensitivity of *pry-1* mutants, two of them (*spp-1* and *ard-1*) affected only the stress response but not the lifespan. These results show that *pry-1* utilizes overlapping subsets of genes in distinct processes. Overall, these findings demonstrate that PRY-1 interacts with a diverse set of conserved factors involved in processes such as protein modification, protein homeostasis, DNA replication, DNA repair, gene expression, and the cell cycle to control essential biological events in *C. elegans*.

## Materials and methods

### Strains

Animals were maintained at 20°C on standard nematode growth media (NGM) plates seeded with OP50 *Escherichiacoli* bacterial strains as described by Brenner (1974). Worm strain information can be found in Supplementary Table S1.

### RNAi

RNAi mediated gene silencing was performed using a protocol previously published by our laboratory (Ranawade *et al.* 2018). Plates were seeded with *E.coli* HT115 expressing either dsRNA specific to candidate genes or empty vector (L4440). Synchronized gravid adults were bleached, and eggs were plated. Vulva or seam cell phenotypes were analyzed in young adults.

Ahringer RNAi library was the source of bacterial clones (Kamath *et al.* 2003). Initially, GO term searches (in 2017; geneontology.org file “gene_ontology.obo” pulled from the version control on May 3 08:44:20 2017 UTC) had identified 36 upregulated genes associated with reproductive structure development. Of these, 26 clones were present in the RNAi library and tested. The remaining clones were either absent or did not grow. Subsequent GO analysis (performed in 2020) revealed a larger set of upregulated genes (52). Since the work on the initial 26 genes had advanced significantly by this time, additional genes were not tested.

### Vulva phenotype and VPC induction analysis

Muv and protruding vulva (Pvl) phenotypes were scored in adults at plate level. Animals with multiple ventral protrusions (pseudovulvae) were termed as Muv and those with a single prominent protrusion as Pvl. VPC induction was determined during the L4 stage under a Nomarski microscope (Seetharaman *et al.* 2010) L4 larvae were mounted on glass slides containing 2% agar pad and the anesthetic sodium azide (1 mM). In a wild-type worm, each of the three VPCs, P5.p, P6.p, and P7.p, are induced to form the vulva (hence the induction score three, one for each precursor). The vulval progeny invaginate and fuse selectively giving rise to a “Christmas tree” appearance during the mid-to-late L4 stage. Animals with more than three induced VPCs (Muv class) show multiple distinct invaginations and are assigned induction scores of greater than three.

### Lifespan analysis

All lifespan analysis was done following adult-specific RNAi treatment using a protocol described previously (Mallick *et al.* 2020). Animals were grown on NGM OP50 seeded plates till late L4 stage after which they were transferred to RNAi plates. Plates were then screened daily for dead animals and surviving worms were transferred every other day till the progeny production ceased. Censoring was done for animals that either escaped, burrowed into the medium, showed a bursting of intestine from the vulva or formed a bag of worms (larvae hatches inside the worm and the mother dies). Data from the lifespan experiments are combined and represented by the Kaplan-Meier survival plot coupled with log-rank (Mantel-Cox) test (to get the statistics of average survival time) (Amrit *et al.* 2014).

### Stress assay

Oxidative stress experiments were performed by exposing animals to 100 mM paraquat (PQ) for 1 and 2 h using a published protocol (Li *et al.* 2008). Final working concentrations were made in M9 buffer. At least 30 animals were tested for each strain in each replicate. Mean and standard deviation were determined from experiments performed in duplicate. Animals were considered dead if they had no response following a touch using the platinum wire pick and showed no thrashing or swimming movement in M9. Moreover, dead animals usually had an uncurled body posture compared to the normal sinusoidal shape of worms.

### Body bending and pharyngeal pumping analysis

Rate of body bending per one min and the rate of pharyngeal pumping per 30 s for adults were analyzed over the period of 4 days (Collins *et al.* 2008). Individual hermaphrodites were analyzed for these phenotypes under the dissecting microscope by placing them on OP50 culture plates. Pharyngeal pumping was assessed by observing the number of pharyngeal contractions for 30 s. For body bending assessment, animals were stimulated by tapping once on the tail of the worm using the platinum wire pick where one body bend corresponded to one complete sinusoidal wave of the worm. Only animals that moved throughout the duration of one min were included in the analysis.

### Molecular biology

RNA was extracted from synchronized L3 and day-1 adult animals. Protocols for RNA extraction, cDNA synthesis and qPCR were described earlier (Ranawade *et al.* 2018). Briefly, total RNA was extracted using Trizol (Thermo Fisher, USA), cDNA was synthesized using the SensiFast cDNA synthesis kit (Bioline, USA), and qPCR was done using the SYBR green mix (Bio-Rad, Canada). Primers used for qPCR experiments are listed in Supplementary Table S1.

### Nomarski fluorescent microscopy

Animals were anesthetized using 10 mM Sodium Azide and mounted on glass slides with 2% agar pads and covered with glass coverslips. Images were captured using Zeiss Apotome microscope and Zeiss software. Fluorescence of *hsp-4::GFP, hsp-60::GFP*, *sod-3::GFP* and *daf-16p::DAF-16::GFP* was examined by analyzing the degree of GFP intensity. Quantification of GFP fluorescence pixel densities was performed with ImageJ^™^ (https://imagej.nih.gov/ij/).

### Statistical analyses

Statistics analyses were performed using Sigma Plot software 11, CFX Maestro 3.1, and Microsoft Office Excel 2016. For lifespan data, survival curves were estimated using the log-rank (Mantel-Cox) test and differences among groups were assessed using the log-rank test. qPCR data were analyzed using Bio-Rad CFX Maestro 3.1 software. For all other assays, data from repeat experiments were pooled and analyzed together and statistical analyses were done using GraphPad Prism 8. *P*-values less than 0.05 were considered statistically significant.

## Results

### Reproductive structure development genes are misregulated in the *pry-1* mutant

**Figure 1:**
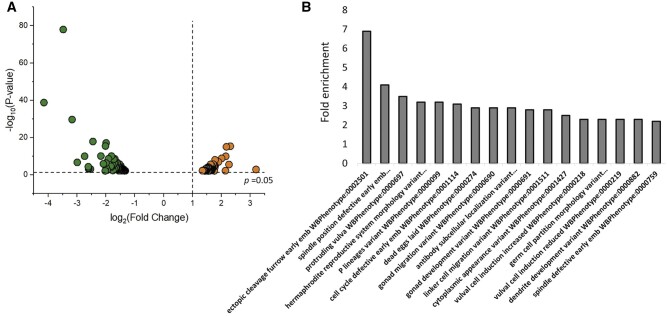
*pry-1* mutant transcriptome is enriched with genes involved in reproductive structure development. (A) Volcano plot showing the 149 differentially expressed genes, with *p <* 0.05, linked to reproductive structure development in *pry-1* mutant animals (Supplementary Table S2). The dotted line on the *x*-axis corresponds to log_2_ fold change of 1 and the one on the *y*-axis shows *p*-value of 0.05. Orange and green dots represent significantly upregulated and downregulated genes, respectively. (B) Phenotype-enrichment analysis of genes shown in (A). Not all categories are listed. See Supplementary Table S4 for a complete list.

Given that *pry-1* is involved in diverse processes, we aimed to identify its interacting genes that might act in a tissue or process-specific manner. The initial work focused on vulval development, a tissue where *pry-1* plays an essential role but very little is known about the downstream target genes. We used GO enrichment analysis (http://geneontology.org/ and Wormbase release WS258) to filter differentially expressed genes associated with “reproductive structure development” (GO:0048608) in the *pry-1* mutants and identified 149 genes (Supplementary Table S2). Among them, 52 and 97 genes were upregulated and downregulated, respectively, in the *pry-1* mutant transcriptome (Ranawade *et al.* 2018) (Figure 1, A and B, Supplementary Table S2). GO term analysis showed significant enrichment (FDR *p<* 0.05) of processes such as cellular component organization or biogenesis (69), anatomical structure development (61), metabolic process (57), regulation of transcription (27), cell cycle (24), and nervous system development (21) (Supplementary Table S3). When examined for molecular functions, we observed enrichment in categories such as protein binding activity (64), DNA binding activity (27), RNA binding activity (20), hydrolase activity (26), signaling receptor binding activity (8), and protein kinase binding activity (7) (Supplementary Table S3). Many genes were found to be associated with cellular components, including the nucleus (74), protein-containing complexes (65), cytoplasm (52), integral components of the membrane (20), cytoskeleton (17), and nuclear chromosomes (12) (Supplementary Table S3). This suggests that *pry-1* plays an essential role in regulating the expression of diverse sets of genes.

Another type of analysis involved enrichment of tissues and phenotypes linked to misregulated genes (https://wormbase.org/tools/enrichment/tea/tea.cgi, Angeles-Albores *et al.* 2016). The results showed significant enrichment of tissues, such as neurons and P-cell lineages (Supplementary Table S4). The genes were also significantly associated with phenotypes such as Pvl, vulval cell induction increased or decreased, and hermaphrodite reproductive system morphology variants (Supplementary Table S4).

### RNAi knockdown of a subset of reproductive structure development genes suppresses the *pry-1* Muv phenotype

**Figure 2:**
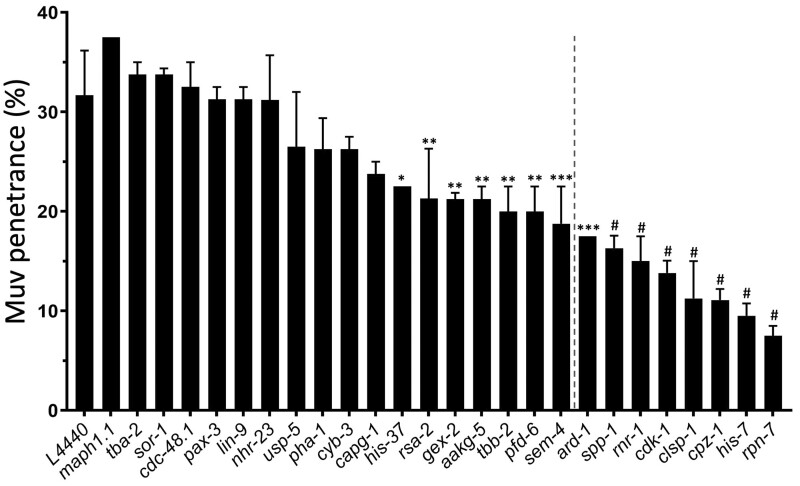
Quantification of the Muv phenotype following RNAi knockdown of 26 upregulated genes in *pry-1(mu38)* animals. Data represent the mean of two replicates (*n* > 40 animals in each replicate) and error bars represent the standard deviation. For eight of the genes, located on the right of the dotted vertical line, Muv penetrance was lower than the mean ± 2x standard deviation of the control (L4440). Statistical analyses were done using one-way ANOVA with Dunnett’s *post hoc* test and significant differences are indicated by stars (*): * (*p <* 0.05), ** (*p <* 0.01), *** (*p <* 0.001), # (*p <* 0.0001).

We evaluated the role of the upregulated genes in *pry-1*-mediated vulval development. To this end, a subset (26 of 52 genes, 50%) was experimentally tested by RNAi to determine the effect of these genes on the Muv phenotype of *pry-1(mu38)* animals (see *Materials andMethods*, Figure 2). As mentioned previously, adult *pry-1* mutant hermaphrodites exhibit a Muv phenotype owing to the constitutive activation of canonical WNT signaling (Gleason *et al.* 2002). Knockdown of 15 of the genes by RNAi significantly suppressed ectopic pseudovulvae in *pry-1* mutants (*p<* 0.05) (Figure 2). For eight of these, the penetrance of the Muv phenotype was lower than the mean ± 2 standard deviations (95% confidence interval) of control RNAi-treated animals (Figure 2); therefore, we designated them as *pry-1* suppressors.

All eight genes, except one (*spp-1*), have homologs in higher eukaryotes including humans, suggesting their important roles in conserved biological processes. Four of the suppressors encode proteins that possess or regulate enzymatic activities, specifically acting as oxidoreductases (*ard-1* and *rnr-1*), a protease (*cpz-1*), and a regulatory subunit of a proteasome complex (*rpn-7*). Two of the suppressors, *clsp-1* and *cdk-1*, are involved in protein phosphorylation. *cdk-1* is possibly necessary for all cell divisions in *C. elegans* (Boxem 2006). *his-7* is a member of the human H2A family of histones that function in DNA repair and gene expression, and *spp-1* is a homolog of the human gene encoding Saposin-like protein that plays a role in immunity (Table 1).

**Table 1:** List of the eight suppressor genes, their mammalian homologs, associated GO-biological processes, and function in *C. elegans*

Gene	Mammalian homolog	GO-biological process	Function in *C. elegans*
*spp-1*	Human SAPLIPs (saposin-like proteins, saposin-B type domain-containing protein)	Pathogenesis; innate immune response; defense response to Gram-negative/positive bacterium; and transmembrane transport	Channel activity
*clsp-1*	Human CLSPN (claspin)	Mitotic G2 DNA damage checkpoint; activation of protein kinase activity; and mitotic DNA replication checkpoint	Anaphase-promoting complex binding activity; part of the Ataxia telangiectasia and Rad3-related (ATR)/ATL-1 DNA replication checkpoint pathway
*ard-1*	Human HSD17B10 (hydroxysteroid 17-beta dehydrogenase 10)	Oxidation-reduction process	Dehydrogenase with NADP binding domain
*rpn-7*	Human PSMD6 (proteasome 26S subunit, non-ATPase 6)	Proteasome-mediated ubiquitin-dependent protein catabolic process	Proteasome 26S complex component; ATP-dependent degradation of ubiquitinated proteins
*cpz-1*	Human CTSZ (cathepsin Z)	Vulval development; proteolysis; embryo development ending in birth or egg hatching; gonad morphogenesis; ecdysis; collagen and cuticulin-based cuticle	Predicted to have cysteine-type endopeptidase activity
*his-7*	Human H2AX (histone H2A type 2-B)	DNA repair; chromatin organization; chromatin silencing; and regulation of transcription	Predicted DNA binding activity
*cdk-1*	Human CDK1 (cyclin-dependent kinase 1)	Cell cycle; cell division; and protein phosphorylation	Serine/threonine kinase activity
*rnr-1*	Human RRM1 (ribonucleotide reductase catalytic subunit M1)	DNA replication; metabolic process; deoxyribonucleotide biosynthetic process; and oxidation-reduction process	Ribonucleoside-diphosphate reductase activity

**Figure 3:**
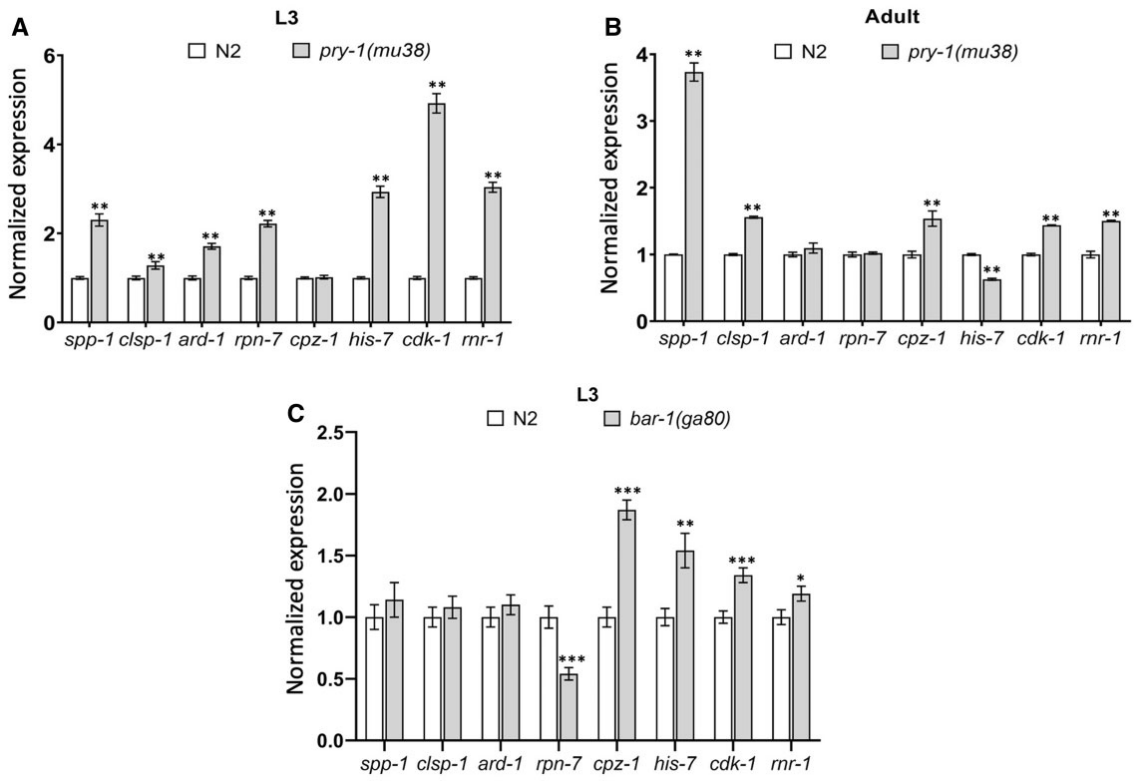
Expression levels of suppressor genes determined by qPCR in *pry-1* and *bar-1* mutants. Normalized expression of suppressor genes in *pry-1(mu38)* L3 larvae and adults (A, B) and *bar-1(ga80)* L3 larvae (C). Each data represents the mean of two replicates and error bars the standard error of means. Significance was calculated using Bio-Rad software (one-way ANOVA) and significant differences are indicated by stars (*): * (*p <* 0.05), ** (*p <* 0.01), *** (*p <* 0.001).

To investigate the expression of the suppressor genes in the *pry-1(mu38)* strain, we performed qPCR experiments at the L3 stage when VPCs undergo division to produce vulval progeny. Earlier, the transcriptome profiling revealed that all genes were upregulated in the L1 larval stage (Ranawade *et al.* 2018). The pattern was the same in L3 animals, except for *cpz-1*, which was unchanged (Figure 3A). We also carried out qPCR analysis in day-1 adults and found that five of the suppressor genes continued to be expressed at significantly high levels. Of the remaining three, *ard-1* and *rpn-7* were unchanged and *his-7* was downregulated (Figure 3B). Thus, most of the suppressor genes are negatively regulated by *pry-1* in L3 larvae and young adults. The results also suggest that *pry-1* regulates some of the genes in a stage and tissue-specific manner. Thus, it could be that *cpz-1* levels are upregulated in vulval cells but not reflected by whole animal qPCR analysis or that its temporal requirement in vulval cells is different. More work is needed to examine these possibilities. Overall, these results support the key roles of suppressor genes downstream of *pry-1* in vulva formation and their potential involvement in mediating *pry-1* function in adults.

**Figure 4:**
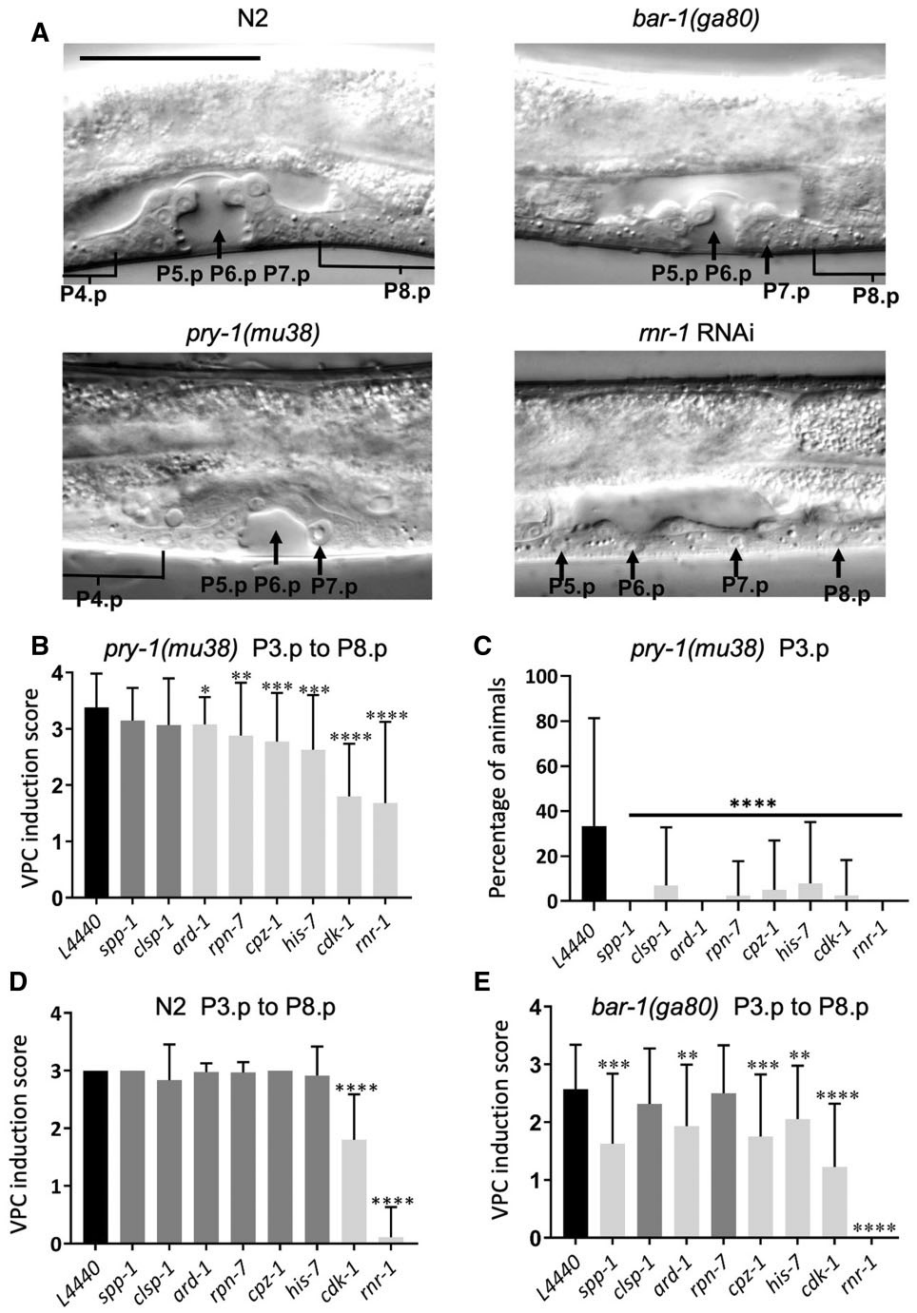
VPC induction analysis following RNAi knockdown of suppressor genes. (A) Representative images of N2, *pry-1(mu38)*, *bar-1(ga80)*, and *rnr-1(RNAi)* animals at the mid-L4 stage. Arrows in N2, *pry-1(mu38)*, and *bar-1(ga80)* animals point to invaginations formed by the progeny of three VPCs (P5.p, P6.p, and P7.p) and to uninduced VPCs in *rnr-1(RNAi)* animal. Not all VPCs and their progeny are shown. Parts of P4.p and P8.p daughter nuclei, where visible, are indicated by half U-shaped lines. Scale bar is 50 µm. (B–E) Panels B, D, and E show average VPC induction (P3.p to P8.p) whereas panel C shows percentage of animals with induced P3.p. Black bars represent control RNAi (L4440), and gray and white represent data that are statistically insignificant and significant, respectively. (B) Knockdown of *ard-1, rpn-7, cpz-1, his-7, cdk-1*, and *rnr-1* significantly reduced average VPC induction in *pry-1(mu38)* animals. (C) Same as B, except that the percentage of animals with induced P3.p is plotted. (D) Knockdown of *cdk-1* and *rnr-1* significantly reduced average VPC induction in N2 animals. (E) Knockdown of *spp-1, ard-1, cpz-1, his-7, cdk-1*, and *rnr-1* significantly reduced average VPC induction in *bar-1(ga80)* animals. In all cases, data shown in panels B–E represent a cumulative of two replicates (*n* > 30 animals in total for each condition, also see Table 2) and error bars represent the standard deviation. Statistical analyses were done using one-way ANOVA with Dunnett’s *post hoc* test and significant differences are indicated by stars (*): * (*p* < 0.05), ** (*p <* 0.01), *** (*p <* 0.001), **** (*p <* 0.0001). Multiple comparison tests were also performed for data in panels B, C, and D using one-way ANOVA for genes that also showed effect in N2 and the results are listed as follows: *cdk-1* RNAi *vs. pry-1(mu38); cdk-1* RNAi (*p* > 0.998), *cdk-1* RNAi *vs. bar-1(ga80); cdk-1* RNAi (*p* = 0.061), *pry-1(mu38); cdk-1* RNAi *vs bar-1(ga80); cdk-1* RNAi (*p* = 0.061), *rnr-1* RNAi *vs pry-1(mu38); rnr-1* RNAi (*p* < 0.0001)****, *rnr-1* RNAi *vs bar-1(ga80); rnr-1* RNAi (*p* = 0.999), *pry-1(mu38); rnr-1* RNAi *vs bar-1(ga80); rnr-1* RNAi (*p* < 0.0001)****.

To understand the cellular basis of Muv suppression, the VPC induction pattern was investigated. In control RNAi treatments, *pry-1(mu38)* animals showed an average VPC induction of 3.4 ± 0.6 (*n* = 29), which is higher than the N2 control (Table 2). The increase was mainly due to P3.p, and to a lower extent P4.p and P8.p, being ectopically induced. The RNAi knockdown of all eight genes suppressed the P3.p defect in *pry-1* mutant animals (Figure 4C, Table 2) (see *Materials and Methods* for details). For six of the genes (*ard-1, rpn-7, cpz-1, his-7, cdk-1*, and *rnr-1. cdk-1* and *rnr-1*) the average VPC induction was also reduced (Figure 4, A and B and Table 2).

**Table 2:** Vulval induction analysis in mutants and RNAi-treated animals

Genotype	RNAi target	% Induced VPCs						% Over induced	VPC induction score	*p*-value	N
		P3.p	P4.p	P5.p	P6.p	P7.p	P8.p				
N2	*Control*	0.0	0.0	100.0	100.0	100.0	0.0	NA	3 ± 0	—	40
	*ard-1*	0.0	0.0	100.0	97.7	100.0	0.0	NA	2.9 ± 0.1	0.360	44
	*cdk-1*	0.0	0.0	47.5	55.0	77.5	0.0	NA	1.8 ± 0.8	<0.001	40
	*clsp-1*	0.0	0.0	92.9	97.6	92.9	0.0	NA	2.8 ± 0.6	0.100	42
	*cpz-1*	0.0	0.0	100.0	100.0	100.0	0.0	NA	3 ± 0	NA	39
	*his-7*	0.0	0.0	97.2	97.2	97.2	0.0	NA	2.9 ± 0.5	0.314	36
	*rnr-1*	0.0	0.0	2.8	5.6	2.8	0.0	NA	0.1 ± 0.5	<0.001	36
	*rpn-7*	0.0	0.0	100.0	100.0	96.7	0.0	NA	2.9 ± 0.1	0.270	30
	*spp-1*	0.0	0.0	100.0	100.0	100.0	0.0	NA	3 ± 0	NA	43
*pry-1(mu38)*	*Control*	31.0	3.4	100.0	100.0	100.0	10.3	37.9	3.4 ± 0.6	—	29
	*ard-1*	0.0	7.9	100.0	97.4	97.4	7.9	15.8	3.1 ± 0.5	0.020	38
	*cdk-1*	2.5	5	65	32.5	72.5	0	10	1.8 ± 0.9	<0.001	40
	*clsp-1*	7.0	7.0	95.3	93.0	88.4	16.3	27.9	3.1 ± 0.8	0.068	43
	*cpz-1*	5.0	5.0	97.5	90.0	75.0	5.0	15.0	2.8 ± 0.9	<0.001	40
	*his-7*	7.9	0.0	89.5	73.7	84.2	10.5	18.4	2.7 ± 0.9	<0.001	38
	*rnr-1*	0.0	9.1	54.5	38.6	56.8	9.1	9.1	1.7 ± 1.4	<0.001	44
	*rpn-7*	2.4	7.1	92.9	85.7	83.3	16.7	23.8	2.9 ± 0.9	0.008	42
	*spp-1*	0	12.5	100	100	97.5	7.5	15	3.2 ± 0.6	0.096	40
*bar-1(ga80)*	*Control*	0.0	0.0	78.6	95.2	83.3	0.0	NA	2.5 ± 0.8	—	42
	*ard-1*	0.0	0.0	59.1	70.5	63.6	0.0	NA	1.9 ± 1.0	0.002	44
	*cdk-1*	0.0	0.0	40.0	32.5	50.0	0.0	NA	1.2 ± 1.1	<0.001	40
	*clsp-1*	0.0	0.0	70.5	86.4	75.0	0.0	NA	2.3 ± 0.9	0.181	44
	*cpz-1*	0.0	0.0	58.3	52.8	63.9	0.0	NA	1.7 ± 1.1	<0.001	36
	*his-7*	0.0	0.0	66.7	61.1	77.8	0.0	NA	2.0 ± 0.9	0.008	36
	*rnr-1*	0.0	0.0	0.0	0.0	0.0	0.0	NA	0	<0.001	36
	*rpn-7*	0.0	0.0	68.4	94.7	91.7	0.0	NA	2.5 ± 0.8	0.690	38
	*spp-1*	0.0	0.0	51.9	55.6	55.6	0.0	NA	1.6 ± 1.2	<0.001	27

Muv phenotype is indicated by % overinduced. *N* refers to the total number of animals examined from all batches combined. NA, not applicable.

We also examined the effects of suppressors on wild-type vulval development. RNAi experiments indicated no significant reduction in VPC induction for any of the genes except *cdk-1* and *rnr-1* (Figure 4, A and D and Table 2). *cdk-1* and *rnr-1* RNAi resulted in 39% and 96.3% reductions in VPC induction, respectively, when compared to that in controls, suggesting that both genes play essential roles in vulva formation. These data, together with expression studies, support our conclusion that all of the suppressor genes act genetically downstream of *pry-1* to regulate vulval development. In addition, it is possible that *cdk-1* and *rnr-1* act in a pathway parallel to *pry-1*.

### Genetic interactions between suppressor genes and *bar-1/β-catenin*

Since PRY-1 is a component of the canonical WNT signaling, we investigated whether any of the suppressor genes function as downstream effectors of the pathway during vulval development. To this end, we used the β-catenin homolog BAR-1, which is negatively regulated by PRY-1 (Eisenmann *et al.* 1998; Gleason *et al.* 2002). Mutations in *bar-1* cause some of the VPCs to remain uninduced. In a *bar-1* null mutant, *bar-1(ga80)*, P3.p and P4.p usually adopt an F (fused) fate. The frequency of the F fate is much lower for the remaining VPCs (12–36% in each case), as they are mostly induced to form the vulva (Eisenmann *et al.* 1998; Eisenmann and Kim 2000). The VPC induction analysis following the RNAi knockdown experiments revealed that for six of the candidate genes—*spp-1*, *ard-1*, *cpz-1*, *his-7*, *cdk-1*, and *rnr-1—*the VPC induction defect of *bar-1(ga80)* was significantly enhanced. *cdk-1* and *rnr-1* RNAi had the most severe effects, and the induction was reduced by 52% and 100%, respectively. In contrast, *clsp-1* and *rpn-7* did not affect VPC induction (Figure 4E and Table 2). We also performed qPCR to examine the levels of suppressor genes in *bar-1(ga80)* animals, which showed that whereas four exhibited increased expression during the L3 stage (*cpz-1*, *his-7*, *cdk-1*, and *rnr-1*), one was reduced (*rnr-7*), and the remaining three were unchanged (*spp-1, clsp-1*, and *ard-1*) (Figure 3C). These results, together with interaction experiments involving *pry-1*, are most consistent with the possibility of *rpn-7* acting genetically downstream of the *pry-1-bar-1* pathway to regulate VPC induction. The remaining genes might mediate *pry-1* function in a *bar-1*-independent manner.

### Suppressor genes influence *pry-1*-mediated nonvulval processes

PRY-1 plays crucial roles in multiple developmental and post-developmental processes; therefore, we examined the involvement of Muv suppressors in all or subsets of PRY-1 nonvulval functions. The phenotypes examined included an increased seam cell number, molting defect, low brood size, developmental delay, stress sensitivity, increased expression of chaperones (*hsp-4/BiP/GRP78*, *hsp-6/HSP70*, and *hsp-60/HSP60*), and short lifespan (Ranawade *et al.* 2018; Mallick *et al.* 2019a, 2020). The partial or complete loss of function of many of the suppressor genes is known to cause defects similar to that of *pry-1* mutants. These include *ard-1* RNAi leading to sterile progeny and developmental delay (Simmer *et al.* 2003; Sönnichsen *et al.* 2005), *rpn-7* and *cpz-1* RNAi animals showing molting defects (Hashmi *et al.* 2004; Frand *et al.* 2005), *clsp-1* RNAi leading to reduced germ line cell proliferation and increased expression of mitochondrial chaperones (Yoneda *et al.* 2004; Ceron *et al.* 2007), and *his-7* RNAi causing slow growth, larval arrest, and extended dauer survivability of *aak-2* mutants (Lehner *et al.* 2006; Ceron *et al.* 2007; Xie and Roy 2012).

To test whether the suppressor genes participate in *pry-1*-mediated developmental processes outside the vulva system, we first examined the seam cells. During seam cell development, *pry-1* promotes asymmetric cell division, and *pry-1* mutants show increased cell numbers. RNAi of the eight genes did not result in a change in the seam cells in both *pry-1* mutant and wild-type animals (Supplementary Figure S1), suggesting that none of these genes play a role in *pry-1*-mediated signaling in generating seam cells.

**Figure 5:**
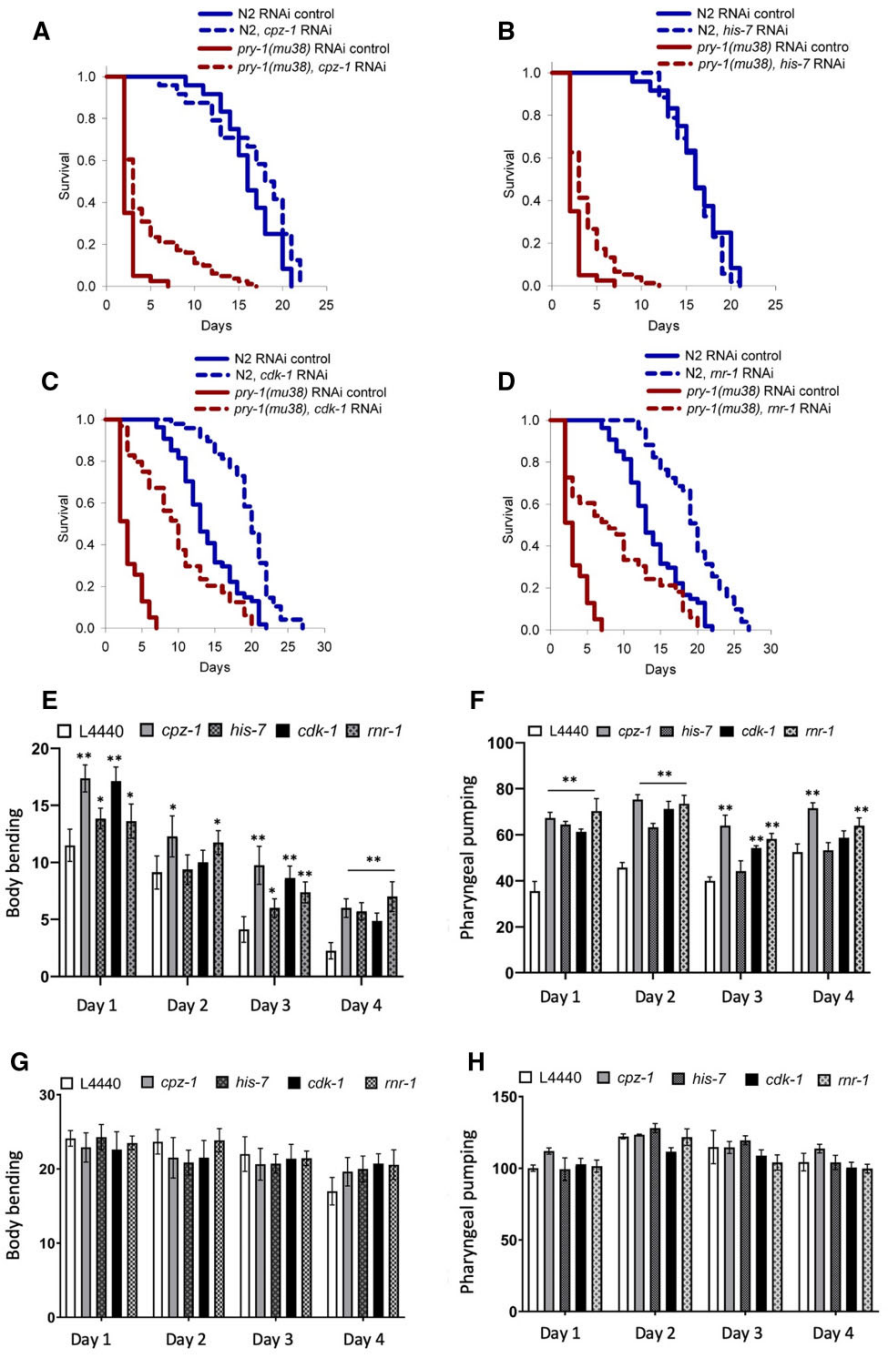
Knockdown of suppressor genes rescue lifespan, body bending and pharyngeal pumping defect of *pry-1* mutants. (A–D) RNAi knockdown of *cpz-1, his-7, cdk-1*, and *rnr-1* in N2 and *pry-1* mutants (also see Supplementary Figure S2). (A–D) See *Materials and Methods* section and Table 3 for lifespan data and statistical analyses. (E, F) Bar graphs showing the rates of body bending and pharyngeal pumping of *pry-1* mutants over a period of 4 days following RNAi of *cpz-1, his-7, cdk-1, and rnr-1*. (G, H) Bar graphs showing the rates of body bending and pharyngeal pumping of N2 animals. (E–H) Data represent the mean of two replicates (n > 10 animals per replicate) and error bars represent the standard deviation. Statistical analyses for panels E–H were done using one-way ANOVA with Dunnett’s *post hoc* test for each day and significant differences are indicated by stars (*): * (*p <* 0.05), ** (*p <* 0.01).

Next, we investigated whether suppressor genes interact with *pry-1* to regulate aging. Recent work from our group demonstrated the essential role of *pry-1* in mediating lifespan maintenance in animals (Mallick *et al.* 2020). RNAi of the suppressor genes in *pry-1(mu38)* mutants from the L4 stage revealed that *cpz-1, his-7, cdk-1*, and *rnr-1* caused a significant extension of the mean lifespan (Figure 5, A–D and Table 3, Supplementary Figure S2). Interestingly, two of these, *cdk-1* and *rnr-1*, also extended the lifespan in wild-type animals, demonstrating their essential function. However, in both cases, increases in the mean lifespan in a *pry-1* mutant background (203% and 170% for *cdk-1* RNAi and *rnr-1* RNAi, respectively) were much higher compared to those observed in wild-type (19.5% and 18.8% for *cdk-1* RNAi and *rnr-1* RNAi, respectively) (Figure 5, C and D; Table 2). The effects of all four genes on body bending and pharyngeal pumping were also examined. We observed that knockdown resulted in significant improvements in both physiological markers of aging in *pry-1* mutants but not in wild-type animals (Figure 5, A–D). Overall, these results allow us to suggest that *cpz-1*, *his-7, cdk-1* and *rnr-1* act downstream of *pry-1* in restricting the lifespan of animals.

**Table 3:** Mean, median, and maximum lifespan of N2 and *pry-1(mu38)* animals following control (empty vector L4440) and gene specific RNAi

Genotype	Treatment	Mean lifespan (day)	Median lifespan (day)	Maximum lifespan (day)	N	*p*-value
N2	L4440	16.37 ± 0.64	16	21	51	—
*pry-1(mu38)*	L4440	3.25 ± 0.26	3	7	79	—
	*spp-1 RNAi*	2.54 ± 0.19	2	8	80	ns
	*clsp-1 RNAi*	4.32 ± 0.31	4	9	80	ns
	*ard-1 RNAi*	3.54 ± 0.45	2	13	79	ns
	*rpn-7 RNAi*	4.07 ± 0.25	4	10	79	ns
	*cpz-1 RNAi*	4.67 ± 0.43	3	17	81	<0.001
	*his-7 RNAi*	4.62 ± 0.26	3	12	75	<0.001
	*cdk-1 RNAi*	9.86 ± 0.68	10	20	64	<0.001
	*rnr-1 RNAi*	8.79 ± 1.12	8	20	76	<0.001
N2	*cpz-1 RNAi*	16.92 ± 0.97	18	22	45	ns
*pry-1(mu38)*		4.67 ± 0.43	3	17	81	<0.001
N2	*his-7 RNAi*	16.09 ± 0.35	16	21	51	ns
*pry-1(mu38)*		4.62 ± 0.26	3	12	75	<0.001
N2	*cdk-1 RNAi*	19.56 ± 0.54	20	27	48	<0.001
*pry-1(mu38)*		9.86 ± 0.68	10	20	64	<0.001
N2	*rnr-1 RNAi*	19.45 ± 0.60	20	27	51	<0.001
*pry-1(mu38)*		8.79 ± 1.12	8	20	76	<0.001

In each case, lifespan data are presented as the cumulative of two replicates (see *Materials and Methods* section). N, number of animals examined; ns, not significant.

**Figure 6:**
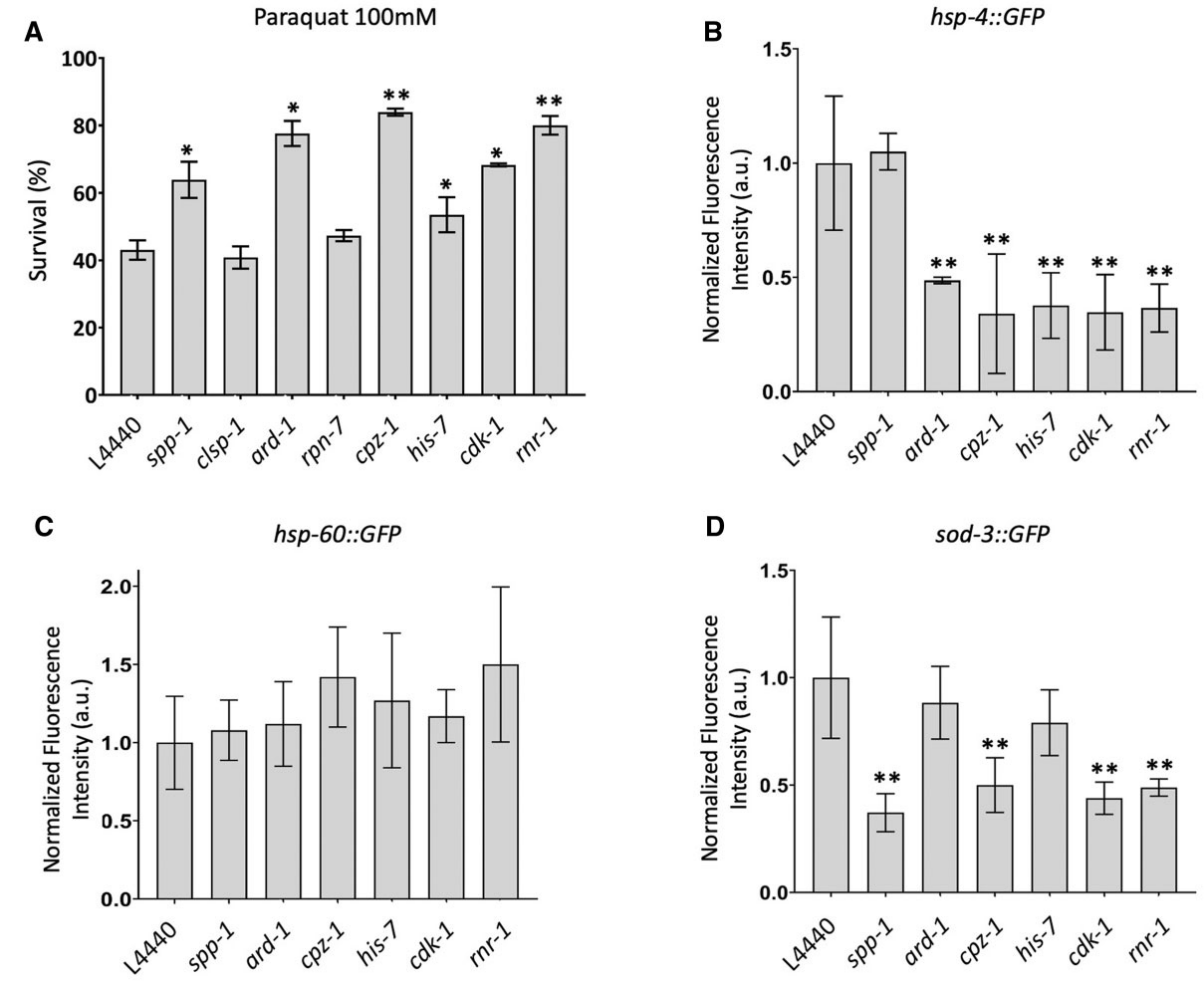
*cpz-1, his-7, cdk-1*, and *rnr-1* regulate stress sensitivity in *pry-1* mutants. (A) Survivability of *pry-1(mu38)* animals following RNAi knockdown of suppressor genes. The animals were treated with 100 mM PQ solution for 1 h. Data represent mean of two replicates (n > 30 animals). (B) Quantification of fluorescence intensity using *hsp-4::GFP* marker in *pry-1* mutants following RNAi knockdown of *spp-1*, *ard-1*, *cpz-1, his-7, cdk-1*, and *rnr-1*. (C) Same as (B), except that fluorescence reporter is *hsp-60::GFP* (D) Same as B, except that fluorescence reporter is *sod-3::GFP*. Data represent the mean of two replicates (n > 15 animals per replicate). Error bars represent the standard deviation. Statistical analyses were done using one-way ANOVA with Dunnett’s post hoc test for each day and significant differences are indicated by stars (*): * (*p <* 0.05), ** (*p <* 0.01).

In addition to its role in aging, *pry-1* is necessary for maintaining the expression of stress response signaling genes (Mallick *et al.* 2020). This led us to examine whether the knockdown of suppressors would decrease the lethality of *pry-1* mutants caused by acute exposure to the nonspecific oxidative stress inducer PQ. Except for *clsp-1* and *rpn-7*, RNAi targeting the suppressor genes reduced the stress sensitivity in *pry-1* mutants (Figure 6A). Interestingly, *ard-1* and *cpz-1* RNAi conferred PQ resistance in wild-type animals as well (Supplementary Figure S3), suggesting crucial roles for both of these genes in maintaining PQ sensitivity in animals. These data suggest that *pry-1* inhibits *spp-1, his-7, cdk-1*, and *rnr-1* to regulate oxidative stress responses in animals. More work is needed to determine whether *ard-1* and *cpz-1* also interact with *pry-1* or act in a parallel pathway.

We investigated whether downregulating the expression of six PQ-responsive genes in *pry-1* mutants could lower cellular stress (Supplementary Figure S4, A and B). This was done using a set of stress response reporters. *pry-1* mutants exhibit increased expression of *hsp-4::GFP* (endoplasmic reticulum unfolded protein response chaperone) and *hsp-60::GFP* (mitochondrial unfolded protein response chaperone) (Supplementary Figure S4B) (Mallick *et al.* 2020). Another stress response reporter that is sensitive to oxidative stress, *sod-3::GFP* (sodium dismutase), was also analyzed. Although *pry-1* mutants do not affect *sod-3::GFP* fluorescence, muscle-specific overexpression of *pry-1* causes an increase in *sod-3* levels (Mallick *et al.* 2020). RNAi targeting all but *spp-1* caused a significant reduction in GFP fluorescence in *hsp-4::GFP* animals (Figure 6B). A similar knockdown in the *sod-3::GFP* strain revealed that the fluorescence was strongly suppressed in the case of *spp-1*, *cpz-1*, *cdk-1*, and *rnr-1* (Figure 6D). There was no significant change in *hsp-60::GFP* expression levels (Figure 6C). Overall, these results show that while there are differences in individual chaperon responses, all six suppressors are involved in *pry-1*-mediated processes and function to maintain the expression of multiple stress-responsive genes.

## Discussion

In this study, we analyzed a set of genes that are upregulated in *pry-1* mutants and associated with “reproductive structure development.” The genes belong to GO categories that include metabolic processes, transcriptional regulation, and mitotic cell cycle. Among the 26 genes tested, RNAi for eight (*spp-1*, *clsp-1*, *ard-1*, *rpn-7*, *cpz-1*, *his-7*, *cdk-1*, and *rnr-1*) suppressed the Muv phenotype of *pry-1* mutant animals with a threshold of the mean ± 2 standard deviations (95% confidence interval). The ectopic P3.p induction defect was also suppressed in all cases. Further gene expression studies and genetic interactions with a null allele of *bar-1* revealed that whereas all of the suppressors function downstream of *pry-1* to regulate vulva formation, only *rpn-7* is involved in *pry-1-bar-1* signaling. In addition, we found that *cdk-1* and *rnr-1* have essential roles because their downregulation caused defects in VPC induction in wild-type animals.

All eight suppressor genes regulate fundamental cellular processes, such as protein phosphorylation (*cdk-1*) and kinase activation (*clsp-1*), protein breakdown (*rpn-7* and *cpz-1*), DNA replication (*rnr-1*), transcription (*his-7*), mitochondrial oxidation and reduction (*ard-1*), and channel activity (*spp-1*) (Table 1). This is also supported by data showing that *clsp-1*, *his-7*, and *cdk-1* transcripts are enriched in germ line cells (Han *et al.* 2017), and *spp-1*, *ard-1*, *cpz-1*, and *rnr-1* are expressed in the neurons, hypodermis, vulva, gonad, intestine, and muscles (Hashmi *et al.* 2004; Alper *et al.* 2007; Hunt-Newbury *et al.* 2007; Srinivasan *et al.* 2008; Keith *et al.* 2016). Therefore, it is not surprising that perturbations in their function result in multiple phenotypes.

**Figure 7:**
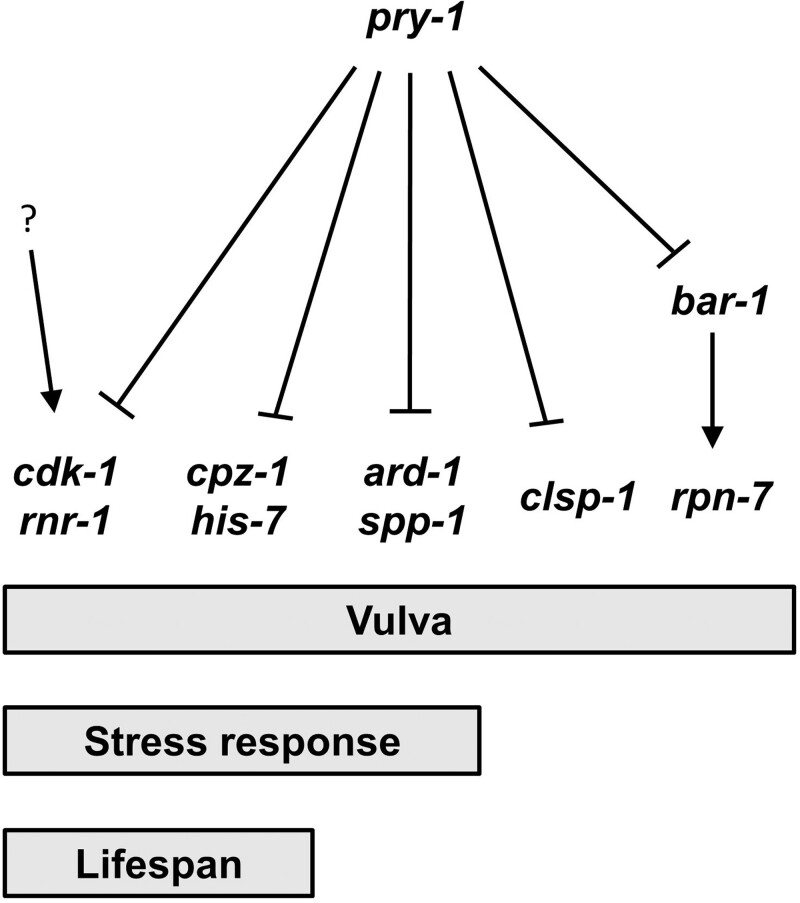
A schematic diagram showing biological processes mediated by *pry-1* and eight suppressor genes. The genetic relationship is based on the expression and functional data described in this study. While all genes are involved in *pry-1*-mediated vulval development, only six affect stress response and four lifespan. Question mark (?) indicates that *cdk-1* and *rnr-1* may also be regulated in a *pry-1*-independent manner.

Whereas our work provides the first evidence for the genetic interactions between *pry-1* and these eight genes (see schematic in Figure 7), some of the genes were previously reported to play roles in vulva formation. Specifically, the disruption of *clsp-1* and *ard-1* causes a Pvl phenotype (Simmer *et al.* 2003; Ceron *et al.* 2007). *cpz-1* localizes to the developing vulva, and *cpz-1* RNAi results in defective vulval morphology (Hashmi *et al.* 2004). *cdk-1* regulates *lin-12/Notch* in a cell cycle-dependent manner (Nusser-Stein *et al.* 2012; Weinstein *et al.* 2015). The remaining four genes, *spp-1*, *rpn-7*, *his-7*, and *rnr-1* had no reported function in the vulva system.

In addition to studying vulval development, we investigated the role of the suppressor genes in *pry-1*-mediated post-developmental events. *spp-1, rpn-7*, *cdk-1*, and *rnr-1* are known to be involved in aging. Specifically, *rpn-7* and *cdk-1* are both required for *glp-1*-mediated lifespan extension (Ghazi *et al.* 2007; Seidel and Kimble 2015), and *spp-1* and *rpn-7* are required for the longevity of *daf-2* mutants (Murphy *et al.* 2003; Ghazi *et al.* 2007; Anyanful *et al.* 2009). *rnr-1* is downregulated in long-lived *daf-2* and *eat-2* mutants, consistent with the notion that high levels of *rnr-1* decrease the lifespan (Gao *et al.* 2018). These genes have potential roles in the conserved aging pathway; therefore, we analyzed their requirement in *pry-1* signaling. Our results showed that *cpz-1*, *his-7*, *cdk-1*, and *rnr-1* act downstream of *pry-1* to affect the lifespan of animals. These four genes, along with *spp-1* and *ard-1*, also play roles in *pry-1*-mediated stress response maintenance, as observed in the acute PQ assay and heat shock chaperone analysis. Thus, whereas *pry-1* negatively regulates many genes, it appears to utilize overlapping subsets in different events (Figure 7). We also found that similar to vulval development *cdk-1* and *rnr-1* extend the lifespan of wild-type animals, suggesting their essential roles in multiple tissues. Both genes may also act in a *pry-1*-independent manner. The involvement of *spp-1* and *ard-1* in the stress response but not aging is consistent with the results of other studies describing genes that have unique roles in these two processes (Chen *et al.* 2009; Bennett *et al.* 2014; Richman *et al.* 2018).

Another mechanism by which some of the suppressor genes may mediate *pry-1* signaling is by regulating proteostasis. Whereas the loss of proteostasis contributes to aging and age-associated abnormalities, its enhancement promotes lifespan extension and results in suppression of age-related diseases (Labbadia and Morimoto 2014; Uno and Nishida 2016). Consistent with *pry-1*’s role in proteostasis, we have found that the gene is necessary for the maintenance of stress response. In addition, preliminary work in our lab has revealed that *pry-1* mutants have defects in protein folding and protein degradation.

Our data also suggest that the roles of *spp-1* in *pry-1* and *daf-2* signaling pathways are different, although the extent to which these differences are reflected in its molecular function is unknown. Considering that *spp-1* encodes a channel protein, it is possible that blocking its activity inhibits unwanted signaling in *daf-2* and *pry-1* mutant adults leading to improved responses. However, animals lacking *spp-1* function are expected to be more sensitive to infections as this gene is involved in maintaining the innate immune response (Anyanful *et al.* 2009). Future experiments are needed to make any firm conclusions regarding the mechanism through which *spp-1* functions in the stress response and lifespan maintenance.

In conclusion, this study identified a new set of interactors of *pry-1* in *C. elegans* that are involved in a range of cellular events, such as protein homeostasis, signaling, gene expression, and cell proliferation, by regulating the activities and stability of proteins and changes in DNA, such as replication, the damage checkpoint, and repair. Some of the suppressors affect multiple *pry-1*-mediated processes, whereas the others appear to have more restricted roles. All of the genes have mammalian homologs or family members, raising the possibility that their interactions with Axin might be conserved. Future studies hold promise to elucidate the mechanism by which these genes mediate tissue-specific functions of Axin in normal and disease conditions.

## Data availability

Strains are available upon request. The authors affirm that all data necessary for confirming the conclusions of the article are present within the article, figures, and tables.

Supplementary material is available at *G3* online.

## Supplementary Material

jkab430_Supplementary_Table_S1

jkab430_Supplementary_Table_S2

jkab430_Supplementary_Table_S3

jkab430_Supplementary_Table_S4

jkab430_Supplemental_Figures_and_Supplemental_Table_Legends
