# Supplementary material for: Genetic analysis of Caenorhabditis elegans pry-1/Axin suppressors identifies genes involved in reproductive structure development, stress responses, and aging
Source: G3 (Bethesda). 2021 Dec 15;12(2):jkab430. doi: 10.1093/g3journal/jkab430 (PMC9210326; doi:10.1093/g3journal/jkab430)
Supplement: jkab430_Supplemental_Figures_and_Supplemental_Table_Legends [file jkab430_supplemental_figures_and_supplemental_table_legends.docx]

**SUPPLEMENTAL MATERIAL**

**Genetic analysis of *Caenorhabditis elegans pry-1/Axin* suppressors identifies genes involved in reproductive structure development, stress responses, and aging**

Avijit Mallick, Nikita Jhaveri, Jihae Jeon, Yvonne Chang, Krupali Shah, Hannah Hosein, and Bhagwati P. Gupta*

Department of Biology, McMaster University, 1280 Main Street West, Hamilton, ON, L8S4K1, CANADA

**FIGURES**


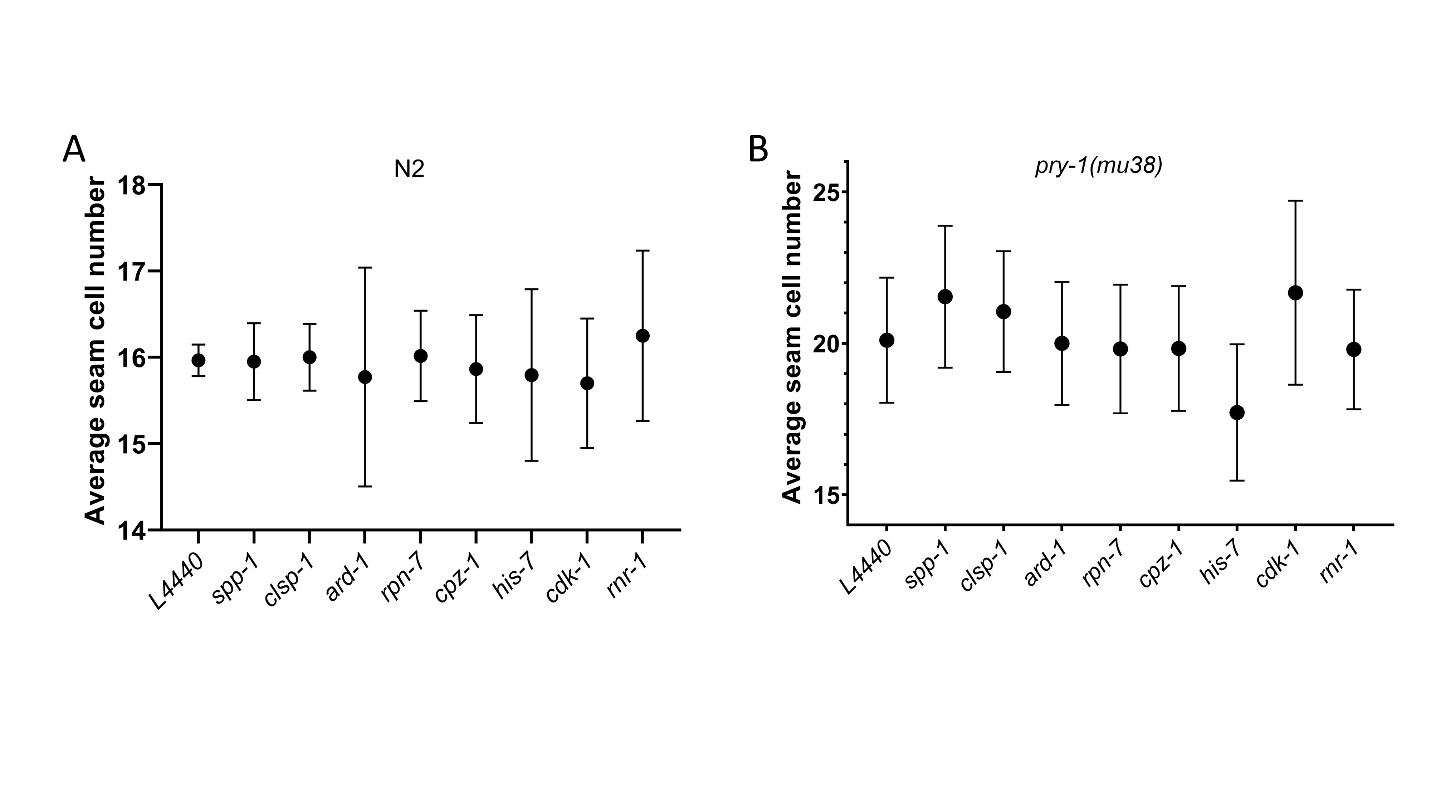


**Figure S1:** Seam analysis in both N2 and *pry-1(mu38)* animals following RNAi of control (L4440) and eight suppressor genes. Data represent mean of two replicates (n > 20 animals) and error bars represent the standard deviation. Statistical analyses were done using one-way ANOVA with Dunnett’s post hoc test.


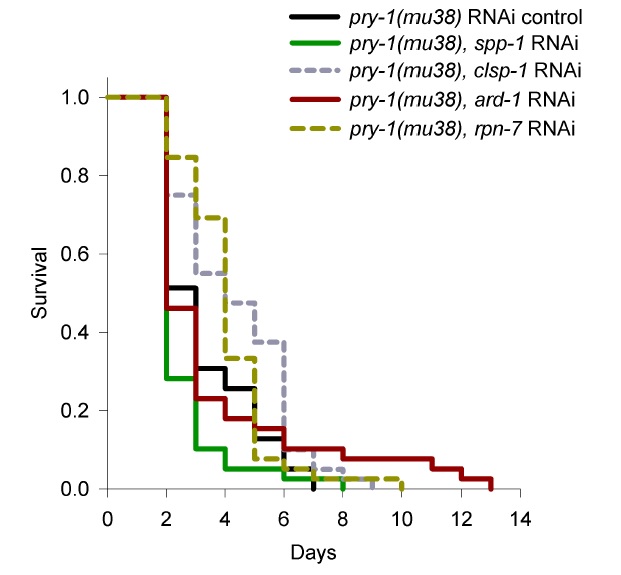


**Figure S2:** Lifespan analysis of *pry-1* mutants following RNAi knockdown of *spp-1, clsp-1, ard-1* and *rpn-7*. See Materials and Methods section and Table 3 for lifespan data and statistical analyses.


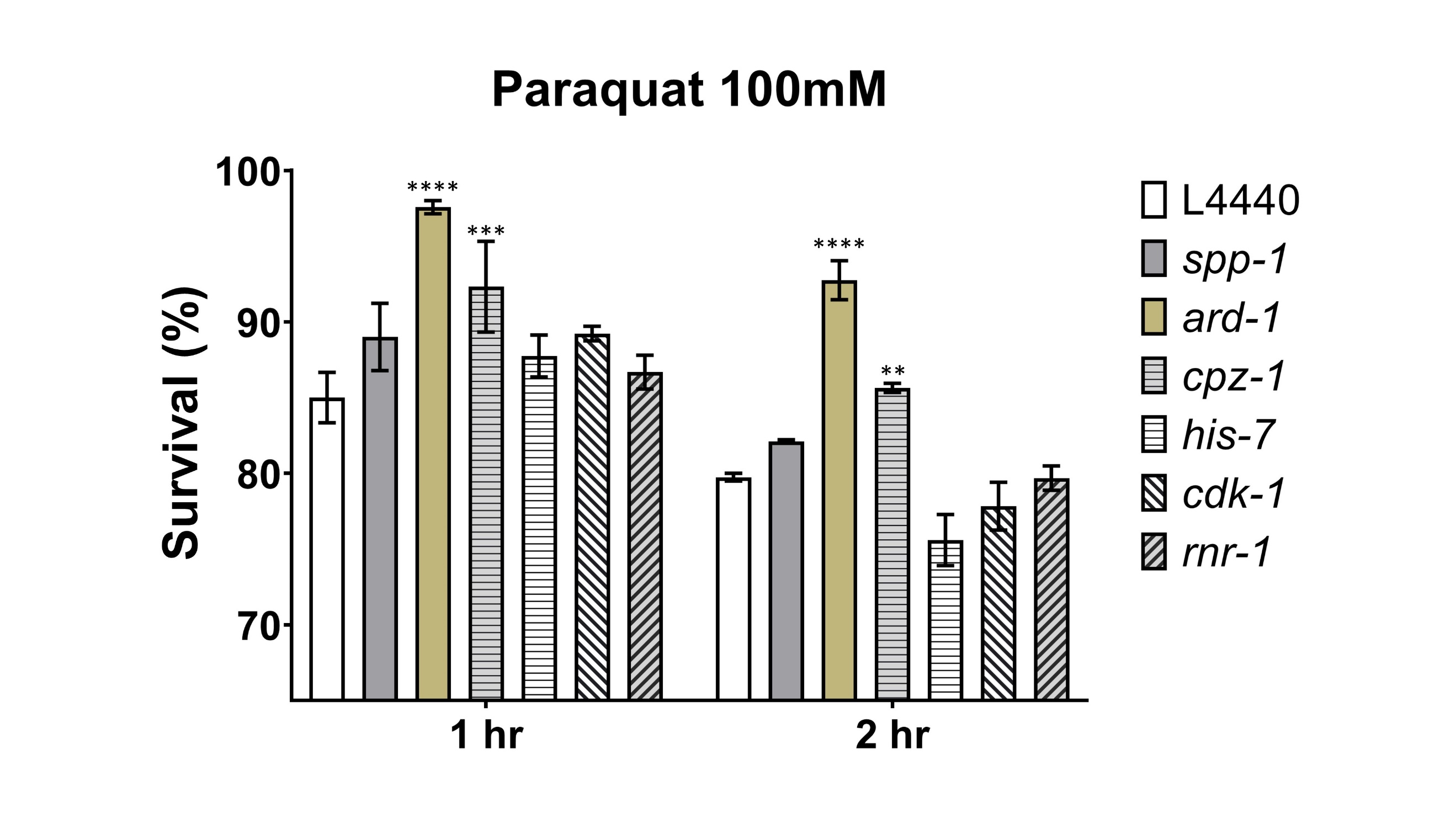


**Figure S3:** Survivability of N2 animals in 100mM PQ solution after 1 hour and 2 hour following RNAi knockdown of genes. Data represent mean of two replicates (n > 40 animals) and error bars represent the standard deviation. Statistical analyses were done using two-way ANOVA with Dunnett’s multiple comparison test for each hour and significant differences are indicated by stars (*): ** (*p* <0.01), *** (*p* <0.001), and **** (*p* <0.0001).


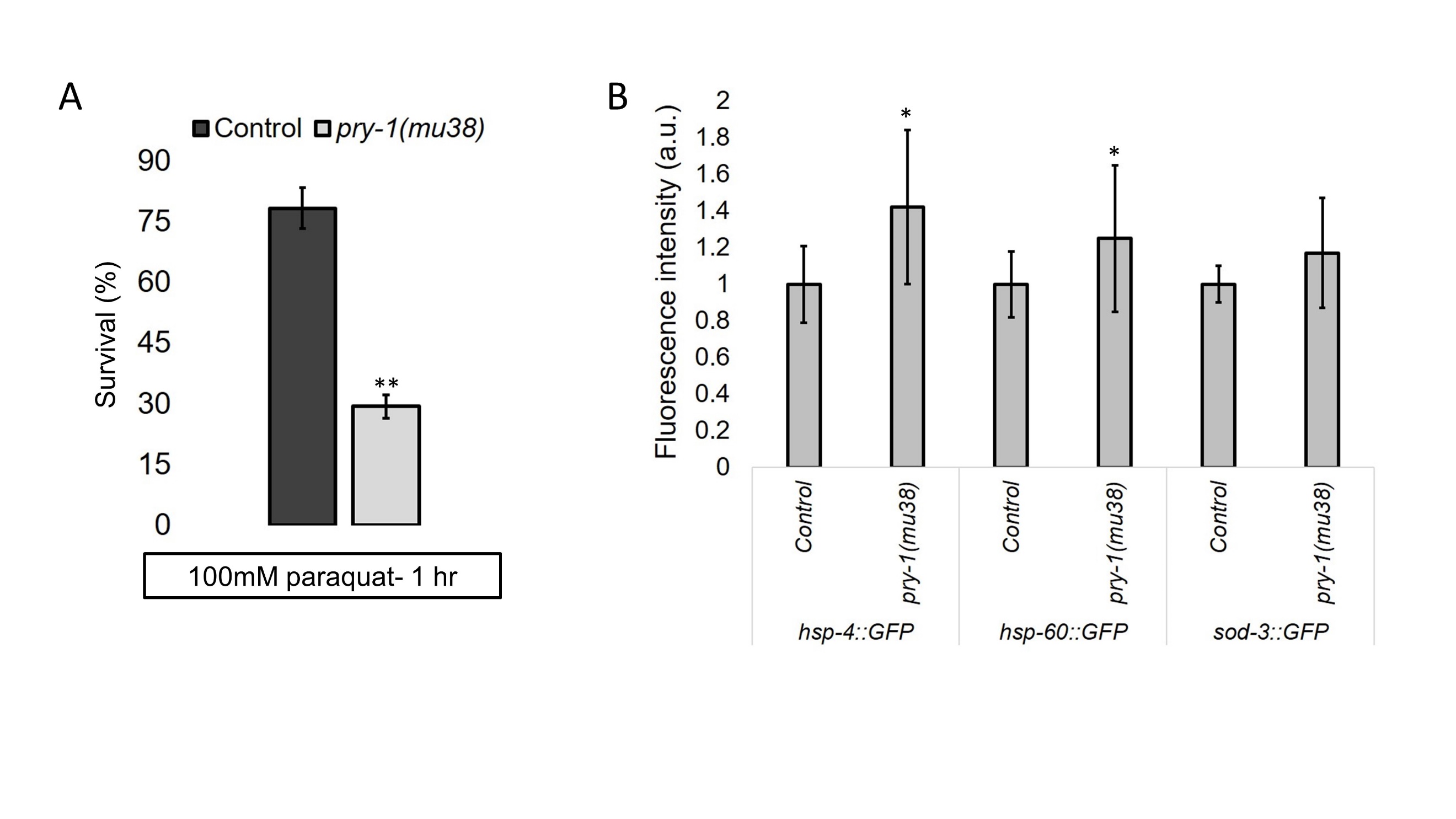


**Figure S4:** *pry-1* mutants are stress sensitive and show increased expression of stress response markers. (A) Stress sensitivity assay of control and *pry-1(mu38)* animals in 100mM PQ solution after 1hr. (B) Quantification of fluorescence intensity using *hsp-4::GFP, hsp-60::GFP* and *sod-3::GFP* marker in control and *pry-1* mutants. Data represent the mean of two replicates (n >30 animals per replicate in A and n >10 animals per replicate in B) and error bars represent the standard deviation. Statistical analyses were done using unpaired student’s t-test with unequal variance significant differences are indicated by stars (*): * (*p* < 0.05), ** (*p* <0.01).

**TABLES:**

**Table S1:** List of strains and primers used in the study.

**Table S2:** List of differentially expressed (DE) genes in *pry-1* mutant transcriptome that are associated with reproductive structure development.

**Table S3:** GO-enrichment analysis of 149 DE genes linked to reproductive structure development.

**Table S4:** Tissue and phenotype-enrichment analysis of 149 DE genes linked to reproductive structure development.
